# Spectral-Spatial Feature Extraction of Hyperspectral Images Based on Propagation Filter

**DOI:** 10.3390/s18061978

**Published:** 2018-06-20

**Authors:** Zhikun Chen, Junjun Jiang, Xinwei Jiang, Xiaoping Fang, Zhihua Cai

**Affiliations:** 1 School of Computer Science, China University of Geosciences, Wuhan 430074, China; chzhikun@163.com (Z.C.); junjun0595@163.com (J.J.); ysjxw@hotmail.com (X.J.); fangxiao_ping@126.com (X.F.); 2 Beibu Gulf Big Data Resources Utilisation Lab, Qinzhou University, Qinzhou 535000, China; 3 Guangxi Key Laboratory of Beibu Gulf Marine Biodiversity Conservation, Qinzhou 535000, China

**Keywords:** propagating filter, hyperspectral image, spectral-spatial feature extraction, image classification

## Abstract

Recently, image-filtering based hyperspectral image (HSI) feature extraction has been widely studied. However, due to limited spatial resolution and feature distribution complexity, the problems of cross-region mixing after filtering and spectral discriminative reduction still remain. To address these issues, this paper proposes a spectral-spatial propagation filter (PF) based HSI feature extraction method that can effectively address the above problems. The dimensionality/band of an HSI is typically high; therefore, principal component analysis (PCA) is first used to reduce the HSI dimensionality. Then, the principal components of the HSI are filtered with the PF. When cross-region mixture occurs in the image, the filter template reduces the weight assignments of the cross-region mixed pixels to handle the issue of cross-region mixed pixels simply and effectively. To validate the effectiveness of the proposed method, experiments are carried out on three common HSIs using support vector machine (SVM) classifiers with features learned by the PF. The experimental results demonstrate that the proposed method effectively extracts the spectral-spatial features of HSIs and significantly improves the accuracy of HSI classification.

## 1. Introduction

Hyperspectral images (HSIs) have many spectral bands and complex spatial structures that contain abundant information [1,2]. Therefore, HSIs are widely applied in areas such as ocean monitoring [3,4], precision agriculture [5,6], forest degradation statistics [7] and military reconnaissance [8]. However, the high-dimensional spectral features of HSIs may cause the Hughes phenomenon [9,10], leading to a decrease in HSI classification accuracy [11,12,13]. Thus, before performing HSI classification, dimensionality reduction (DR) and feature extraction techniques [14,15] are typically used to obtain low-dimensional and discriminative features for classification [16,17].

Many DR models have been utilized to pre-process high-dimensional HSIs, including supervised, unsupervised, and semi-supervised DR methods [18]. Examples of supervised DR methods include linear discriminant analysis (LDA) [19] and nonparametric weighted feature extraction (NWFE) [20]; the unsupervised methods include PCA [21], independent component analysis (ICA) [22], superpixelwise PCA [23]; and the semi-supervised DR methods include semi-supervised discriminant analysis (SDA) [24]. Among these methods, the new optimized feature extracted by the best discriminant vector satisfies the class separability after the samples in high-dimensional feature space are projected to the low-dimensional feature space through the supervised DR model LDA. However, when the data samples between classes are nonlinearly separated in the input space, LDA is expected to fail. The semi-supervised DR technique SDA adds a regularization term to the LDA algorithm to ensure that the local structure between the samples is preserved during feature extraction. The unsupervised DR approach ICA represents HSIs with a relatively small number of independent features; however, ICA is more complicated than PCA, which is a simple, rapid and effective method for unsupervised DR that has been widely used in HSI feature extraction because it can extract the most informative features of HSIs using only a few principal components. However, the drawback of PCA is that it considers each pixel separately, regardless of the pixels’ spatial context information, which has proven to be very effective prior knowledge [25,26,27].

To make better use of the spatial context information, the most commonly adopted strategy is to leverage the filter to extract HSI features. For example, Li et al. [28] developed the PCA-Gabor-SVM algorithm, which improved HSI classification accuracy by combining spatial and spectral information to filter dimensionality-reduced features from PCA. The edge-preserving filtering algorithm (EPF) proposed in [29] utilized PCA to decompose greyscale or colour-guided images, taking advantage of the edge-preserving properties of bilateral filtering and guided filtering. Methods that combine spatial and spectral information obviously enhance the classification performance by preserving the spatial structure. Pan et al. [30] constructed a hierarchical guidance filtering and a matrix of spectral angle distance and iteratively trained classifiers using the integrated learning spatial and spectral information from different scales to achieve good generalization performance. The deep learning method proposed by Zhou et al. [31] achieved very good results by using convolutional filters that learned directly from images by extracting their spectral-spatial features. Wei et al. [32] proposed a hierarchical deep framework called spectral-spatial response that uses a template acquired through Marginal Fisher Analysis and PCA to learn the combination of spectral-spatial features simply.

The aforementioned filters have demonstrated the ability to represent the latent spatial structures embedded in HSIs. However, HSI cross-regional mixing typically exists due to the limited spatial resolution and the complexity of the feature distribution. That is, the filter template, which consists of adjacent pixels centered on the target pixel, not only includes the characteristics of the target features but also a mixture of other features. The cross-regional mixture affects the implementation of smooth filtering or other filtering tasks, leading to fuzzy areas and inefficient features for HSI classification. Shen et al. [33] proposed a multiscale spectral-spatial context-aware propagation filter that extracts the features of hyperspectral images from multiple views to generate spatial-spectral features. The PF addresses the cross-regional mixing problem of HSIs, however a too-large or too-small scale parameter may have a negative impact and is not conducive to the suppression of cross-regional mixing problems. Therefore, this paper proposes a novel spectral-spatial feature extraction method of an HSI based on the PF method, which addresses the cross-regional mixing problem in HSIs effectively.

The structure of this paper is as follows. Section 2 details the proposed method. Experimental results and discussions are given in Section 3, and Section 4 summarizes this paper.

## 2. Proposed Method

### 2.1. Propagation Filter

The PF [34] is a smoothing filter in which the pixel values of an HSI are acquired by

(1) $$O_{s}^{\prime }=\frac{1}{Z_{s}}\sum_{t\in N_{s}}\omega_{s,t}I_{t},$$

where $Z_{s}=\sum_{t\in N_{s}}\omega_{s,t}$ is the normalised factor, $N_{s}$ is the set of neighbouring pixels set, the size of the window size is ((2w+1)×(2w+1)) for the central pixel *s*, $\omega_{s,t}$ is the weight of pixel *t* of the neighbouring pixel set to perform the filtering of pixel *s*, and $I_{t}$ is the pixel value of pixel *t* of the HSI.

Here, $\omega_{s,t}=P\left(s⟶t\right)$ is defined as the weight between pixel *s* and the its adjacent pixel *t* such that t=s. The weight between pixel *s* and itself is $\omega_{s,s}=P\left(s⟶s\right)=1$; otherwise,

(2) $$\omega_{s,t}=\omega_{s,t-1}D\left(t-1,t\right)R\left(s,t\right),$$

where the two distances D(t−1,t) and R(s,t) can be defined by

(3) $$D\left(t-1,t\right)=g\left(∥I_{t-1}-I_{t}∥;\sigma_{\alpha }\right),$$

(4) $$R\left(s,t\right)=g\left(∥I_{s}-I_{t}∥;\sigma_{r}\right),$$

with the function g(.) being the Gaussian function

(5) $$g\left(∥I_{t-1}-I_{t}∥;\sigma_{\alpha }\right)=\exp\left(\frac{-∥I_{t-1}-I_{t}{∥}^{2}}{2\sigma_{\alpha }^{2}}\right),$$

(6) $$g\left(∥I_{s}-I_{t}∥;\sigma_{r}\right)=\exp\left(\frac{-∥I_{s}-I_{t}{∥}^{2}}{2\sigma_{r}^{2}}\right).$$

Without the loss of generality, it is assumed that $\sigma_{\alpha }=\sigma_{r}$ and D(.)=R(.) throughout the this paper.

### 2.2. Spectral-Spatial Feature Extraction Method Based on the PF

**Figure 1:**
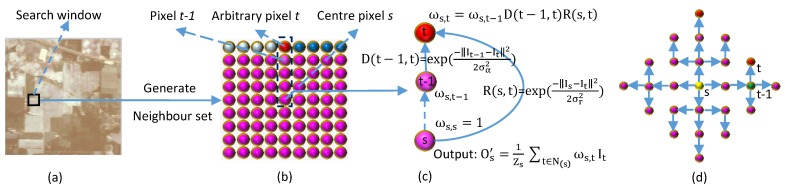
Flow diagram of HSI filtering using the PF. (**a**) Hyperspectral image (**b**) Neighbour pixel set $N_{s}$ (**c**) the calculation of $\omega_{s,t}$ and (**d**) the pattern for performing 2D filtering with *w* = 3 pixels.

As shown in Figure 1a, the cross-region mixture problem is quite common in HSIs. In particular, a lower spatial resolution increases the number of classes. As the ground sample distance increases, there is a potential for objects covered by a given pixel to be mixed [34]. Therefore, this paper presents the spectral-spatial feature extraction of the HSI algorithm based on the advantage that the PF can handle the cross-regional mixture problem [35]. As seen in Equations (1)–(6) and Figure 1b–d, the PF generates a new center pixel using a weighted summation of the neighbouring pixels in the HSI. The adjacent pixel *t*, center pixel *s* and pixel *t*− 1 in the neighbouring pixel set are all the same class, and the weight of pixel *t* is relatively larger. In Figure 1d, pixel *t*− 1 selected is close to the pixel *t* and points to the pixel *t*, where pixel *s* is in yellow, pixel *t* is in red, pixel *t*− 1 is in green. However, when there are mixed pixels in the neighbouring pixel sets, the weight of pixel *t* is smaller. Therefore, the PF ensures that the similar features of the same classes of pixels are enhanced, which suppresses the effects of cross-regional mixed pixels. 

In addition, to improve the performance of the PF for feature extraction in HSIs, PCA is performed before filtering: the HSIs are reduced by PCA, and the redundant information between bands is greatly reduced in the updated pixels. However, although the HSIs lose a small amount of information after PCA, the bands are sorted according to the importance of the information. After the PF process, the increased effects of the important and reduced effects of the less important features are beneficial for feature extraction and in improving the classification accuracy.

**Figure 2:**
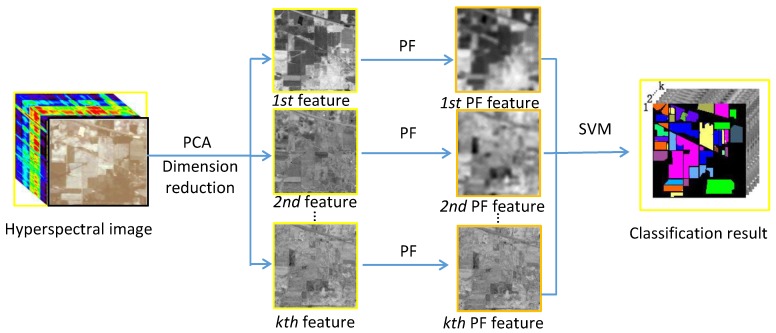
Schematic of the proposed PCA-PF-SVM method.

The specific process is shown in Figure 2. In the first step, PCA is used to reduce the dimensionality and remove the redundant inter-spectral information to obtain the principal components of an HSI. Then, the PCA feature is filtered with the PF. When cross-regional mixing occurs in the image, the filter template reduces or avoids the influence of cross-regional mixed pixels on the object pixel, thereby avoiding or effectively mitigating the effects of cross-regional mixed pixels. Through this technique, the proposed method can accurately extract the reflected spectral-spatial features of the real objects. Finally, to validate the effectiveness of the proposed method, experiments are carried out on HSIs using an SVM classifier trained on the learned spectral-spatial features. Algorithm 1 depicts the proposed HSI spectral-spatial feature extraction model based on the PF.

**Algorithm 1:** Specific flowchart of the spectral-spatial feature extraction algorithm based on the PF.

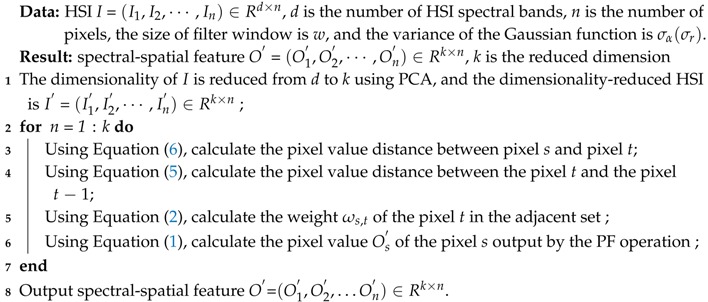



## 3. Experimental Settings

In this paper, the training and testing samples for each HSI dataset were chosen randomly. In the experiments shown in Table 1, 20 label samples were randomly selected for each class as training samples, and the rest were used as test samples to verify the performance of the proposed methods in the three experiments. To verify the classification performance of different methods with sufficient training samples and insufficient training samples, in the experiments shown in Table 5, 10–50 training samples were selected from each class and the rest were used as test samples. For stability, each experiment was performed 10 times; the reported results are the averages.

### 3.1. Dataset Description

Three real HSI sets are used in this paper: the Indian Pines, Salinas and University of Pavia scenes. The Indian Pines image was obtained by the AVIRIS sensor and covers the northern agricultural Indian Pines test site. The image, which includes 16 categories of ground objects, contains 145×145 pixels, and only 200 out of all 224 bands are valid due to water absorption. The spatial resolution is 20 m per pixel, and the spectral range is 0.4 to 2.5 μm. The Salinas image contains 512×217 pixels and includes 16 types of ground objects at a 3.7-m spatial resolution and was acquired by the AVIRIS sensor over the Salinas Valley in California, USA. After removing 20 of the 224 bands due to noise and water absorption, the remaining 204 valid bands were utilized in the experiments. The University of Pavia image was acquired with 610×340 pixels at 1.3-m spatial resolution by the ROSIS Sensor in the city area around the University of Pavia. The image has a spectral range of 0.43 to 0.86 μm with 115 bands, where 12 bands that were noisy or impacted by water absorption were removed, and the remaining 103 bands were used.

### 3.2. Compared Algorithms

In the experiments, the proposed classification method PCA-PF-SVM was compared to other widely used HSI classification methods, including SVM [11], PCA-SVM [36], PCA-Gabor-SVM [28], EPF-SVM [29], HiFi [30], LBP-SVM [37], R-VCANet-SVM [38] and PF-SVM. The parameters used for these methods were the default settings provided in the related literature. The source code for the algorithms was provided by the respective authors. The SVM classifier was based on the Libsvm library [39], and the optimal parameters of the SVM classifier were determined by a fivefold cross validation. The overall accuracy (OA), the average accuracy (AA), and the kappa coefficient are used to evaluate the performance of the methods. The OA indicates the probability that the classification results are consistent with the reference classification results. The AA refers to the mean of the percentage of correctly classified pixels for each class. The kappa coefficient is used for consistency check.

### 3.3. Parameter Sensitivity Analysis

**Figure 3:**
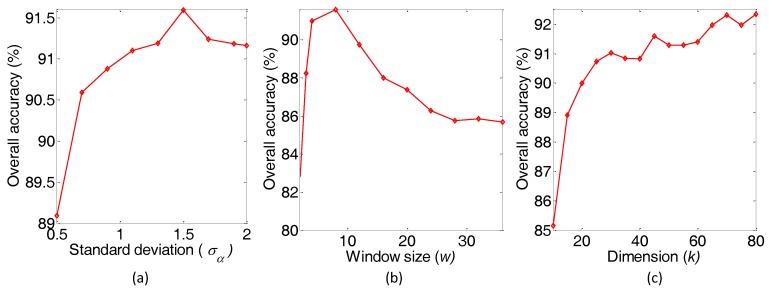
Indian Pines: analysis of the influence of parameters. (**a**) Standard deviation $\sigma_{\alpha }\left(\sigma_{r}\right)$; (**b**) Window size *w* and (**c**) Dimension *k*.

The proposed PCA-PF-SVM method has the following three important parameters: the filtering standard deviation $\sigma_{\alpha }\left(\sigma_{r}\right)$, the filtering window size (w) and the feature dimension (k). To test the influence of the different parameter settings of the proposed model, we conducted extensive experiments were conducted on the Indian Pines scene. As shown in Figure 3a, the best OA, AA and kappa values were achieved when $\sigma_{\alpha }\left(\sigma_{r}\right)=1.5$. In contrast, when $\sigma_{\alpha }\left(\sigma_{r}\right)<1.5$, the accuracies decreased significantly because a small $\sigma_{\alpha }\left(\sigma_{r}\right)$ leads to a smoother image. When $\sigma_{\alpha }\left(\sigma_{r}\right)>1.5$, the classification accuracy remains relatively stable because the ability to suppress bad information improves after the filter parameter reaches a certain value. As shown in Figure 3b, the best OA, AA and kappa values were achieved when w=8. These values are significantly lower when w<8 because considerable important spatial information is lost when the window size is too small. Moreover, the values also decrease when w>8 because the window contains a larger amount of irrelevant information that reduces the effect of the important spatial information and, thus, reduces the classification accuracy. From Figure 3c, OA becomes lager with the increase of PCA dimensions. When the dimension reaches to 45, OA trends to become decrease. In our experiments, k is set to 45 for the tradeoff between the computation complexity and classification accuracy. Therefore, in all of our experiments, the parameters were set as follows: $\sigma_{\alpha }\left(\sigma_{r}\right)=1.5$, w=8 and k=45.

### 3.4. Experimental Results

**Figure 4:**
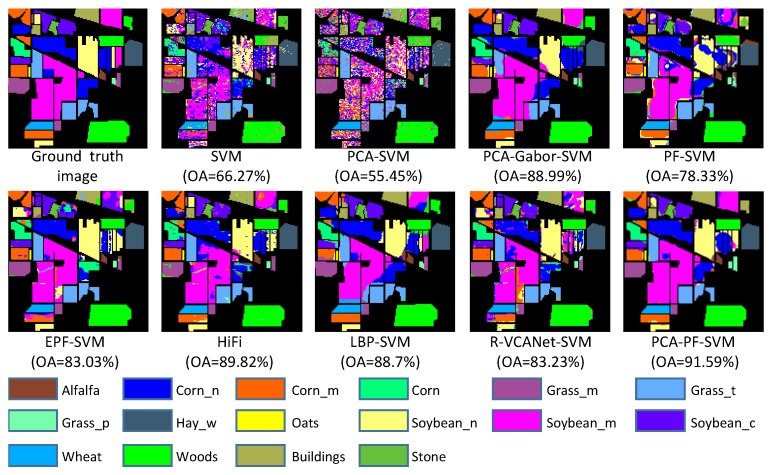
The classification results of the Indian Pines image.

**Figure 5:**
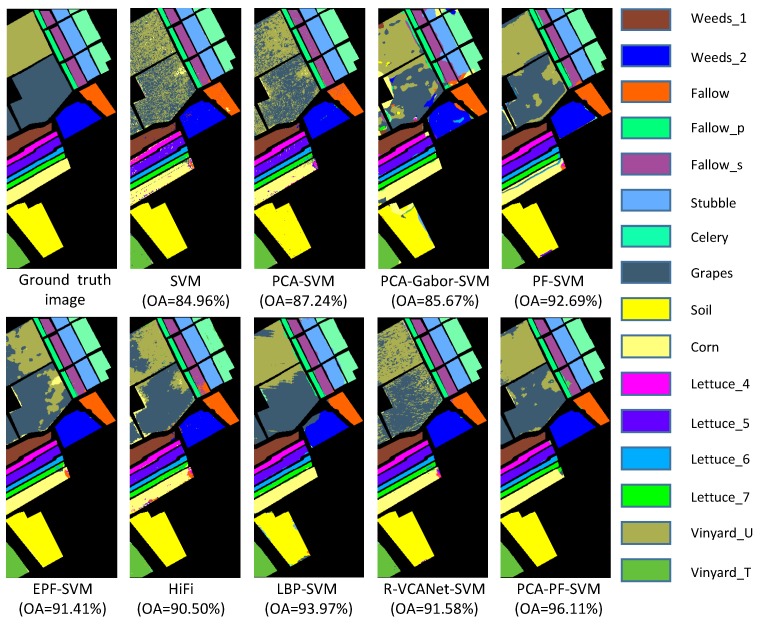
The classification results of the Salinas image.

**Figure 6:**
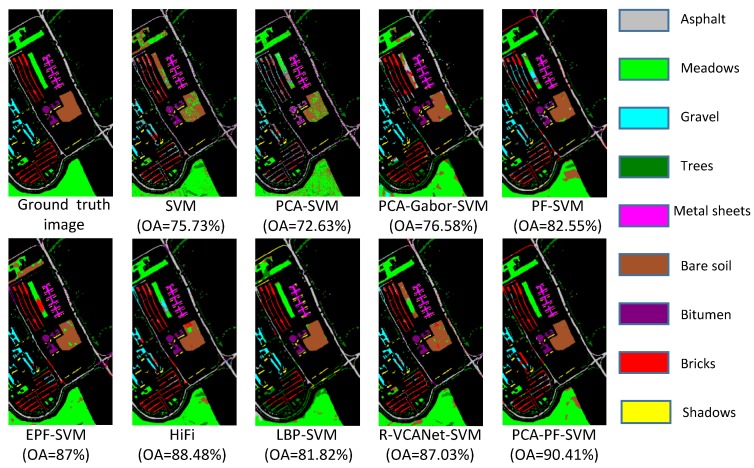
The classification results of the University of Pavia image.

**Figure 7:**
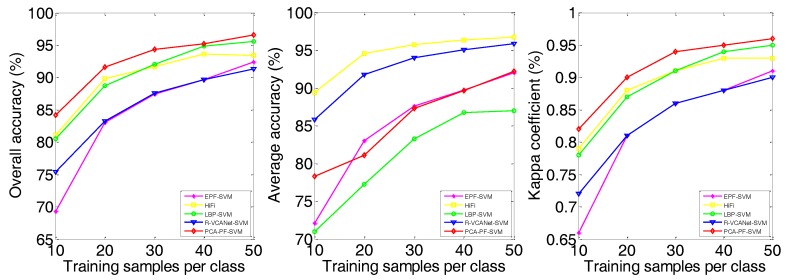
Influence of training samples on Indian Pines dataset.

**Figure 8:**
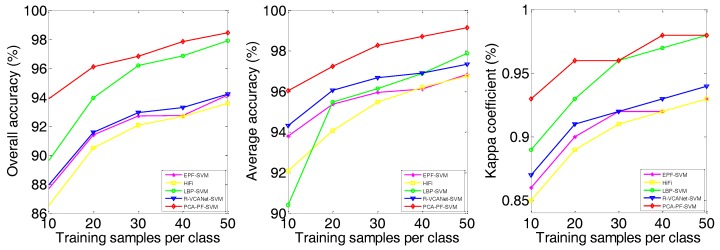
Influence of training samples on Salinas dataset.

**Figure 9:**
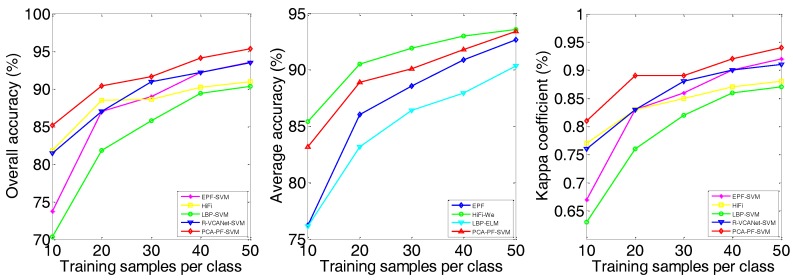
Influence of training samples on University of Pavia dataset.

**Figure 10:**
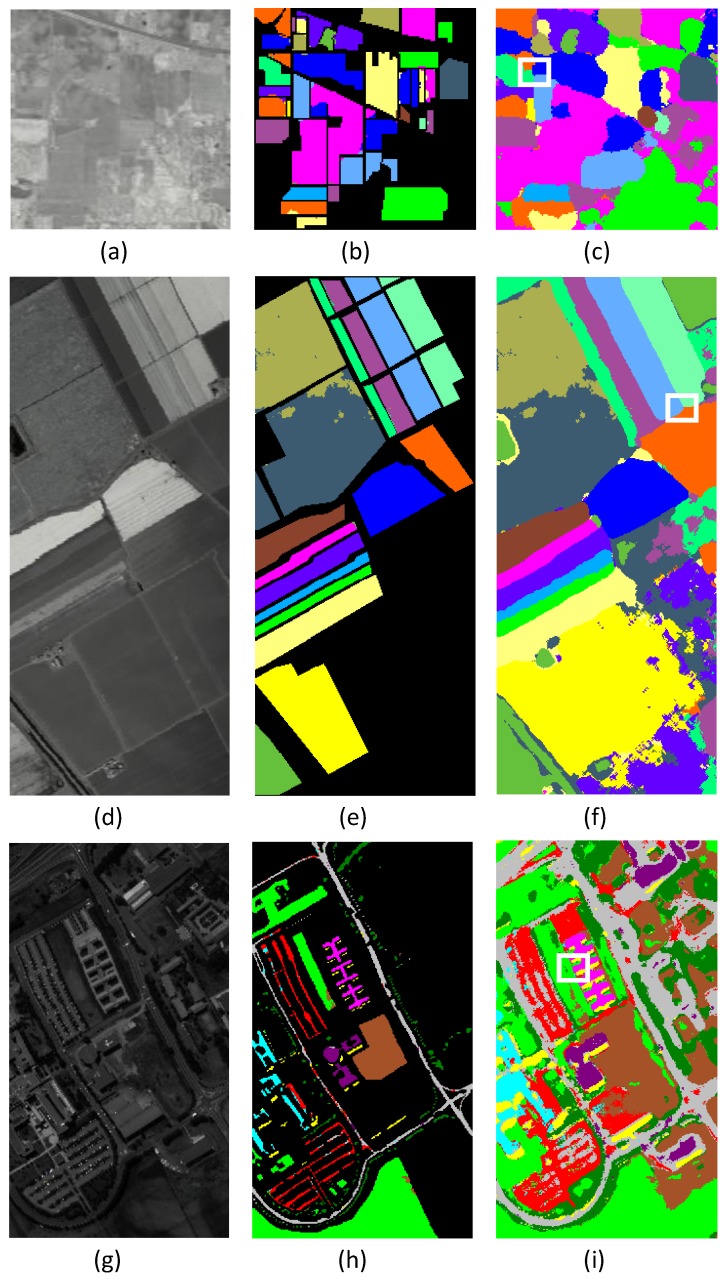
Classification maps of PCA-BF-SVM methods on three datasets. (**a**,**d**,**g**) are false color composite image (R-G-B = band 50-27-17) for Indian Pines , University of pavia and Salinas datasets; (**b**,**e**,**h**) are ground truth classification results image; (**c**,**f**,**i**) are complete classified results image.

**Figure 11:**
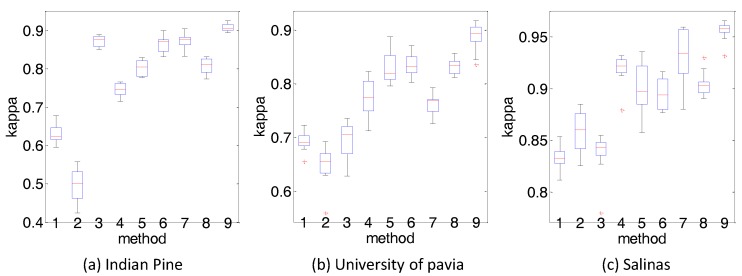
Box plot of kappa of different methods on three datasets. (**a**) Indian Pine (**b**) University of pavia (**c**) Salinas 1. SVM 2. PCA-SVM 3. PCA-Gabor-SVM 4. PF-SVM 5. EPF-SVM 6. HiFi 7. LBP-SVM 8. R-VCANet-SVM 9. PCA-PF-SVM. The center line is the median value, the edges of the box are the 25th and 75th percentiles, the whiskers extend to the most extreme points, and the abnormal outliers are plotted by “+”.

(1) The proposed PCA-PF-SVM method has strong spatial capabilities. According to Figure 4, Figure 5 and Figure 6 and Table 2, Table 3 and Table 4, the PCA-PF-SVM method achieves better OA, AA and kappa values than does the spectral classification method. The OA values based on the proposed PCA-PF-SVM method with respect to the Indian pines, Salinas and University of Pavia datasets are 36.14%, 8.87% and 17.78% higher, respectively, than the OA values based on the PCA-SVM method and 25.32%, 11.15% and 14.68% higher, respectively, than the OA values based on the SVM method. The main reason is that the spectral classification methods do not consider spatial information, while the method proposed in this paper fully considers spatial information. These results verify that the proposed method is effective in spectral-spatial feature extraction.

(2) The results verify that combining PCA and the PF is effective for HSI feature extraction. Figure 4, Figure 5 and Figure 6 and Table 2, Table 3 and Table 4 show that the PCA dimensionality reduction of the HSI does not improve the performance of the SVM classification and may even reduce the classification performance of the SVM. For example, the OA values of the PCA-SVM method for the Indian Pines dataset are lower than those for the SVM method. This result mainly occurs because although the PCA preserves the HSI’s main information, it also loses a small amount of information, thus affecting the SVM classification accuracy. However, the combination of PCA and the PF greatly enhances the performance. The OA values based on the proposed PCA-PF-SVM method for the Indian pines, Salinas and University of Pavia datasets are 13.26%, 3.42% and 7.86% higher, respectively, than are the OA values resulting the PF-SVM method. These experimental results show that it is necessary to apply PCA dimensionality reduction before filtering.

(3) The proposed method is more effective than the other advanced classification methods. As shown in Figure 4, Figure 5 and Figure 6 and Table 2, Table 3 and Table 4, compared with other methods, the PCA-PF-SVM method shows very good performance in terms of OA and kappa. On the Indian Pines, Salinas and University of Pavia datasets, the PCA-PF-SVM method shows more obvious effects than do the HiFi-We, LBP-SVM and R-VCANet-SVM methods. The OA values based on the proposed PCA-PF-SVM method for the Indian Pines, Salinas and University of Pavia datasets are 1.77%, 5.61% and 1.93% higher, respectively, than the OA values based on the HiFi-We method and 2.89%, 2.14% and 8.59% higher, respectively, than the OA values based on the LBP-SVM method and 8.36%, 4.53% and 3.38% higher, respectively, than the OA values based on the R-VCANet-SVM method.

(4) The experimental results demonstrate the robustness of the proposed PCA-PF-SVM method. As shown in Figure 7, Figure 8 and Figure 9 and Table 5, in both scenarios, as the number of training samples varies from 10 to 50, the proposed method achieves the highest OA. Its advantage is especially obvious when the number of training samples is small. For example, when the number of training samples per class is 10, our method has a 3.12–36.31% advantage on the Indian Pines image and a 3.5–20.29% advantage on the Salinas image and a 3.31–23.43% advantage on the University of Pavia image compared to the other methods. This is a highly meaningful result, because it means that a large number of non-labelled samples can be distinguished using a much smaller number of labelled samples, thus greatly improving the work efficiency, which further illustrates the robustness of the proposed method.

(5) These experimental results show that the proposed method is useful for addressing the cross-regional mixture problems of HSIs. In Figure 10, the complete classified maps and ground truth maps obtained by PCA-PF-SVM are presented. The proposed method achieves better results on the cross-region mixture problem. For cross-region marked by white box in the three figures, PF can reduce cross-region problem, which keep better feature of image and improve further classification accuracy.

(6) Statistical evaluation about the results: To further validate whether the observed increase in kappa is statistically significant, we use paired *t*-test to show the statistical evaluation about the results. T-test is popular in many related works [40,41,42]. We accept the hypothesis that the mean kappa of PCA-PF-SVM is larger than a compared method only if Equation (7) is valid:

(7) $$\frac{\left({\bar{a}}_{1}-{\bar{a}}_{2}\right)\sqrt{n_{1}+n_{2}-2}}{\sqrt{\left(\frac{1}{n_{1}}+\frac{1}{n_{2}}\right)\left(n_{1}s_{1}^{2}\right)+n_{2}s_{2}^{2}}}>t_{1-\alpha }\left[n_{1}+n_{2}-2\right]$$

where ${\bar{a}}_{1}$ and ${\bar{a}}_{2}$ are the means of kappa of PCA-PF-SVM and a compared method, $s_{1}$ and $s_{2}$ are the corresponding standard deviations, $n_{1}$ and $n_{2}$ are the number of realizations of experiments reported which is set as 10 in this paper. Paired *t*-test shows that the increases on kappa are statistically significant in all the three datasets (at the level of 95%), and it can be also observed in Figure 11.

## 4. Conclusions

The motivation for this study was to develop a simple feature extraction method to handle the cross-regional mixed problem of HSIs. The developed method extracts spectral-spatial features via the PF. However, the HSI’s high-dimensional problems affect the PF’s performance to a certain extent. To ensure a real effect, based on the characteristics of the HSI, PCA is used to reduce an images dimensions. Moreover, a combination PCA-PF feature extraction method is proposed. To evaluate the performance of the proposed method, three classical datasets with different complexities of cross-regional mixing problems were analyzed, and comparative experiments were also employed. The results show that the proposed method effectively solves the cross-regional mixture problem. In addition, feature extraction method in this paper use NRS and ELM for classification, and compares with PCA-Gabor-NRS and LBP-ELM.As shown in Table 6, classification results show that our method can obtain better results than that of the compared methods.

## Figures and Tables

**Table 1:** Train-Test Distribution of sample for three datasets.

Indian Pines			Salinas			University of Pavia		
Class	Train	Test	Class	Train	Test	Class	Train	Test
Aifalfa	20	26	weeds_1	20	1989	Asphalt	20	18,629
Corn_n	20	1408	weeds_2	20	3706	Meadows	20	2079
Corn_m	20	810	fallow	20	1956	Gravel	20	3044
Corn	20	217	fallow_p	20	1374	Trees	20	1325
Grass_m	20	463	fallow_s	20	2658	Sheets	20	5009
Grass_t	20	710	stubble	20	3939	Soil	20	1310
Grass_p	14	14	Celery	20	3559	Bitumen	20	3662
Hay_w	20	458	Grapes	20	11,251	Bricks	20	927
Oats	10	10	Soil	20	6183	Shadows	20	170
Soybean_n	20	952	Corn	20	3258			
Soybean_m	20	2435	Lettuce_4	20	1048			
Soybean_c	20	573	Lettuce_5	20	1907			
Wheat	20	185	Lettuce_6	20	896			
Woods	20	1245	Lettuce_7	20	1050			
Buildings	20	366	Vinyard_U	20	7248			
Stone	20	73	Vinyard_T	20	1787			

**Table 2:** Classification accuracy of different methods on Indian Pines data set (%).

Class	SVM	PCA-SVM	PCA-Gabor-SVM	PF-SVM	EPF-SVM	HiFi	LBP-SVM	R-VCANet-SVM	PCA-PF-SVM
Aifalfa	55.00	54.35	70.27	12.38	57.78	100.00	46.58	100.00	54.55
Corn_n	52.16	51.32	81.18	67.18	85.80	84.94	89.95	65.41	95.22
Corn_m	63.35	25.22	90.78	77.55	89.35	93.09	86.70	85.31	94.97
Corn	53.33	28.45	82.20	72.53	43.06	87.10	91.85	97.24	91.44
Grass_m	82.80	75.81	97.37	90.89	92.93	92.01	88.72	91.36	72.16
Grass_t	85.91	86.62	96.19	87.59	91.93	97.61	85.70	96.48	100.00
Grass_p	37.14	53.85	45.16	35.00	82.35	100.00	30.00	100.00	18.92
Hay_w	97.89	99.76	88.59	100.00	100.00	99.78	88.49	99.13	100.00
Oats	27.27	38.89	24.39	8.85	100.00	100.00	13.89	100.00	45.45
Soybean_n	57.38	29.14	95.84	68.79	66.32	93.70	74.14	83.61	84.34
Soybean_m	71.57	51.75	87.75	91.33	92.13	78.52	97.06	71.79	95.90
Soybean_c	37.88	36.69	93.13	68.58	52.77	94.24	85.89	87.43	88.51
Wheat	88.14	96.83	77.02	95.81	100.00	99.46	83.12	99.46	95.85
Woods	92.55	93.98	95.49	96.61	96.94	98.23	99.84	95.74	100.00
Buildings	39.31	53.67	90.20	74.44	88.99	93.99	95.87	95.36	72.58
Stone	95.77	87.65	76.04	34.45	87.95	100.00	78.43	100.00	87.01
OA	66.27 ± 2.46	55.45 ± 4.38	88.99 ± 1.33	78.33 ± 1.69	83.03 ± 1.85	89.82 ± 2.01	88.70 ± 1.93	83.23 ± 1.75	91.59 ± 1.32
AA	64.84 ± 2.28	60.25 ± 5.63	80.73 ± 1.60	67.62 ± 1.52	83.02 ± 3.19	94.54 ± 0.97	77.26 ± 2.58	91.77 ± 0.82	81.06 ± 3.91
kappa	0.62 ± 0.02	0.50 ± 0.04	0.87 ± 0.02	0.76 ± 0.02	0.81 ± 0.02	0.88 ± 0.02	0.87 ± 0.02	0.81 ± 0.01	0.90 ± 0.01

**Table 3:** Classification accuracy of different methods on Salinas data set (%).

Class	SVM	PCA-SVM	PCA-Gabor-SVM	PF-SVM	EPF-SVM	HiFi	LBP-SVM	R-VCANet-SVM	PCA-PF-SVM
weeds_1	98.05	100.00	88.18	98.07	100.00	98.49	99.40	99.90	100.00
weeds_2	99.37	99.43	88.99	99.92	99.89	98.70	99.26	99.84	99.84
fallow	91.22	94.35	82.46	93.93	94.91	99.80	97.92	99.39	100.00
fallow_p	97.68	94.41	73.87	86.13	97.86	97.45	83.89	99.56	91.79
fallow_s	97.00	95.24	81.13	97.62	99.96	88.75	97.28	99.62	99.52
stubble	100.00	99.95	92.22	99.95	99.92	99.59	95.13	99.97	99.97
Celery	99.94	100.00	96.04	98.22	100.00	96.60	94.66	98.17	100.00
Grapes	72.98	76.85	92.01	91.63	82.04	82.13	91.57	78.54	95.28
Soil	98.59	99.00	97.29	99.49	99.48	99.97	99.97	99.26	99.97
Corn	79.39	93.32	64.75	92.48	85.06	87.97	99.04	94.69	97.76
Lettuce_4	93.65	91.02	95.66	95.42	98.21	96.18	98.96	98.76	100.00
Lettuce_5	94.34	91.97	97.63	96.07	100.00	99.48	99.89	100.00	100.00
Lettuce_6	93.37	91.14	84.29	76.19	96.10	97.21	92.64	94.31	98.33
Lettuce_7	92.29	94.26	90.26	99.41	99.20	92.67	95.97	96.86	93.09
Vinyard_U	54.30	58.25	73.37	77.59	73.97	73.17	83.00	85.32	85.01
Vinyard_T	94.44	99.54	94.03	98.59	99.49	96.75	99.17	99.27	95.21
OA	84.96 ± 1.17	87.24 ± 1.73	85.67 ± 1.99	92.69 ± 1.38	91.41 ± 2.29	90.50 ± 1.32	93.97 ± 2.28	91.58 ± 1.09	96.11 ± 0.86
AA	91.04 ± 0.53	92.42 ± 0.93	87.01 ± 1.78	93.80 ± 0.85	95.38 ± 0.85	94.06 ± 0.68	95.48 ± 1.62	96.05 ± 0.40	97.24 ± 0.45
kappa	0.83 ± 0.01	0.86 ± 0.02	0.84 ± 0.02	0.92 ± 0.02	0.90 ± 0.03	0.89 ± 0.01	0.93 ± 0.03	0.91 ± 0.01	0.96 ± 0.01

**Table 4:** Classification accuracy of different methods on University of Pavia data set (%).

Class	SVM	PCA-SVM	PCA-Gabor-SVM	PF-SVM	EPF-SVM	HiFi	LBP-SVM	R-VCANet-SVM	PCA-PF-SVM
Asphalt	87.52	82.14	72.39	85.47	98.05	80.40	84.36	79.96	92.30
Meadows	91.00	90.51	95.96	97.60	97.40	89.74	97.98	83.39	99.47
Gravel	61.72	39.42	75.01	56.17	89.16	82.92	72.93	88.12	84.96
Trees	70.10	79.54	40.27	80.30	96.20	83.64	51.19	96.75	76.68
Sheets	98.42	100.00	88.21	99.25	95.05	99.17	86.32	100.00	99.92
Soil	46.04	53.61	68.69	70.30	64.27	89.72	75.02	93.57	84.80
Bitumen	54.64	32.06	78.94	71.72	58.20	96.79	76.85	99.01	85.61
Bricks	80.23	57.68	80.20	60.79	76.20	92.55	78.43	88.39	79.43
Shadows	100.00	99.35	49.44	83.23	99.89	99.46	45.34	100.00	96.95
OA	75.73 ± 1.64	72.63 ± 3.40	76.58 ± 2.98	82.55 ± 3.41	87.00 ± 2.43	88.48 ± 1.90	81.82 ± 1.68	87.03 ± 1.19	90.41 ± 1.90
AA	76.63 ± 1.43	70.48 ± 2.41	72.12 ± 2.81	78.31 ± 3.34	86.05 ± 2.39	90.49 ± 0.97	74.27 ± 2.19	91.17 ± 0.89	88.90 ± 2.05
kappa	0.69 ± 0.02	0.65 ± 0.04	0.70 ± 0.03	0.78 ± 0.04	0.83 ± 0.03	0.83 ± 0.02	0.76 ± 0.02	0.83 ± 0.01	0.89 ± 0.02

**Table 5:** Classification accuracy using varying numbers of training samples applied to three datasets.

Method	Quality Indexes	Indian Pines					Salinas					University of Pavia				
		Training Samples Perclass					Training Samples Perclass					Training Samples Perclass				
		10	20	30	40	50	10	20	30	40	50	10	20	30	40	50
SVM	OA	57.43	66.27	73.31	75.94	78.66	82.64	84.96	86.42	86.20	87.70	67.02	75.73	78.95	82.30	83.78
	AA	55.87	64.84	69.84	72.67	75.86	88.87	91.04	91.38	91.77	92.75	69.12	76.63	77.69	80.23	81.36
	kappa	0.52	0.62	0.70	0.73	0.76	0.81	0.83	0.85	0.85	0.86	0.59	0.69	0.73	0.77	0.79
PCA-SVM	OA	47.89	55.45	58.47	62.07	66.67	84.47	87.24	88.59	88.37	89.30	61.71	72.63	76.53	77.90	80.41
	AA	53.23	60.25	64.14	67.02	72.15	88.98	92.42	93.89	93.99	94.40	60.60	70.48	74.04	75.29	77.15
	kappa	0.42	0.50	0.53	0.57	0.62	0.83	0.88	0.87	0.87	0.88	0.52	0.65	0.70	0.72	0.75
PCA-Gabor-SVM	OA	76.03	88.99	93.06	94.64	96.09	73.62	85.67	89.29	93.08	94.46	65.51	76.58	81.26	84.30	86.18
	AA	75.90	80.73	86.93	88.79	91.78	76.95	87.01	90.49	93.70	94.91	63.76	72.12	77.19	80.11	83.28
	kappa	0.73	0.87	0.92	0.94	0.96	0.71	0.84	0.88	0.92	0.94	0.57	0.70	0.76	0.80	0.82
PF-SVM	OA	64.77	78.33	84.19	87.84	90.40	88.69	92.69	94.28	95.16	95.46	71.23	82.55	87.62	89.13	91.73
	AA	59.06	67.62	73.27	77.47	82.39	91.24	93.80	95.77	96.42	96.64	68.91	78.31	82.82	83.76	87.38
	kappa	0.61	0.76	0.82	0.86	0.89	0.97	0.92	0.94	0.95	0.95	0.64	0.78	0.84	0.86	0.89
EPF-SVM	OA	69.32	83.03	87.41	89.63	92.41	87.71	91.41	92.70	92.73	94.15	73.76	87.00	88.97	92.19	93.57
	AA	72.06	83.02	87.60	89.74	92.02	93.80	95.38	95.96	96.12	96.85	76.21	86.05	88.56	90.89	92.66
	kappa	0.66	0.81	0.86	0.88	0.91	0.86	0.90	0.92	0.92	0.93	0.67	0.83	0.86	0.90	0.92
HiFi	OA	81.08	89.82	91.65	93.63	93.44	86.53	90.50	92.08	92.67	93.59	81.83	88.48	88.64	90.22	90.94
	AA	89.44	94.54	95.74	96.36	96.72	92.08	94.06	95.47	96.20	96.76	85.40	90.49	91.91	92.99	93.58
	kappa	0.79	0.88	0.91	0.93	0.93	0.85	0.89	0.91	0.92	0.93	0.77	0.83	0.85	0.87	0.88
LBP-SVM	OA	80.49	88.70	92.01	94.85	95.58	89.65	93.97	96.18	96.86	97.91	70.35	81.82	85.75	89.39	90.34
	AA	70.96	77.26	83.29	86.72	87.00	90.41	95.48	96.13	96.88	97.87	66.39	74.27	81.33	84.85	86.41
	kappa	0.78	0.87	0.91	0.94	0.95	0.89	0.93	0.96	0.97	0.98	0.63	0.76	0.82	0.86	0.87
R-VCANet-SVM	OA	75.40	83.23	87.56	89.66	91.33	87.96	91.58	92.93	93.29	94.21	81.47	87.03	90.95	92.18	93.46
	AA	85.82	91.77	94.00	95.05	95.88	94.32	96.05	96.68	96.91	97.34	87.21	92.13	93.51	94.48	95.51
	kappa	0.72	0.81	0.86	0.88	0.90	0.87	0.91	0.92	0.93	0.94	0.76	0.83	0.88	0.90	0.91
PCA-PF-SVM	OA	84.20	91.59	94.32	95.23	96.55	93.91	96.11	96.83	97.84	98.45	85.14	90.41	91.62	94.12	95.34
	AA	78.28	81.06	87.29	89.65	92.22	96.04	97.24	98.28	98.70	99.14	83.17	88.90	88.26	91.80	93.41
	kappa	0.82	0.90	0.94	0.95	0.96	0.93	0.96	0.96	0.98	0.98	0.81	0.89	0.89	0.92	0.94

**Table 6:** Classification Results obtained by PCA-Gabor-NRS, PCA-PF-NRS, LBP-ELM and PCA-PF-ELM.

**Indian Pines**												
**Training Samples Perclass**	**PCA-Gabor-NRS**			**PCA-PF-NRS**			**LBP-ELM**			**PCA-PF-ELM**		
	**OA**	**AA**	**kappa**	**OA**	**AA**	**kappa**	**OA**	**AA**	**kappa**	**OA**	**AA**	**kappa**
10	68.46	61.32	0.65	84.50	76.99	0.83	80.89	89.16	0.79	83.15	90.43	0.81
20	82.56	75.63	0.80	90.82	83.84	0.90	88.37	93.62	0.87	91.44	95.32	0.90
30	88.93	83.28	0.87	93.73	87.69	0.93	92.57	96.09	0.92	94.35	96.81	0.94
40	91.99	87.17	0.91	94.79	89.67	0.94	94.42	96.76	0.94	95.69	97.68	0.95
50	93.71	89.21	0.93	95.72	90.08	0.95	95.76	97.77	0.95	97.08	98.37	0.97
**Salinas**												
**Training Samples Perclass**	**PCA-Gabor-NRS**			**PCA-PF-NRS**			**LBP-ELM**			**PCA-PF-ELM**		
	**OA**	**AA**	**kappa**	**OA**	**AA**	**kappa**	**OA**	**AA**	**kappa**	**OA**	**AA**	**kappa**
10	57.53	55.95	0.54	93.54	95.64	0.93	90.41	92.92	0.89	93.22	96.70	0.92
20	75.74	75.55	0.73	95.97	97.46	0.96	94.90	96.47	0.94	95.96	98.12	0.96
30	87.62	88.11	0.86	96.91	98.24	0.97	96.46	97.84	0.96	96.58	98.49	0.96
40	91.94	92.2	0.91	97.41	98.48	0.97	97.69	98.38	0.97	97.90	98.99	0.98
50	94.85	94.8	0.94	7.93	98.74	0.98	98.02	98.67	97.79	98.40	99.23	0.98
**University of Pavia**												
**Training Samples Perclass**	**PCA-Gabor-NRS**			**PCA-PF-NRS**			**LBP-ELM**			**PCA-PF-ELM**		
	**OA**	**AA**	**kappa**	**OA**	**AA**	**kappa**	**OA**	**AA**	**kappa**	**OA**	**AA**	**kappa**
10	50.86	51.76	0.41	80.73	78.73	0.75	73.98	76.15	0.67	82.18	82.47	0.77
20	63.07	62.57	0.55	89.18	86.87	0.86	82.47	82.9	0.78	89.42	89.09	0.86
30	69.39	67.65	0.62	93.06	91.04	0.91	86.52	86.42	0.82	91.13	91.26	0.88
40	76.64	75.21	0.71	94.48	92.77	0.93	88.83	87.93	0.85	92.69	92.52	0.90
50	82.26	81.09	0.78	95.21	93.73	0.94	90.77	90.36	0.88	94.60	93.42	0.93
